# Neighbourhood socio-economic status and positive affectivity among older residents in Germany: a cross-sectional analysis with data from the Heinz Nixdorf Recall Study

**DOI:** 10.1186/s12877-022-03459-9

**Published:** 2022-11-22

**Authors:** Christina Hartig, Gabriele Bolte, Karl-Heinz Jöckel, Susanne Moebus, Natalie Riedel

**Affiliations:** 1 Institute of Public Health and Nursing Research, Department of Social Epidemiology, University of Bremen, Grazer Straße 4, 28359 Bremen, Germany; 2 Health Sciences Bremen, University of Bremen, Bremen, Germany; 3 Institute of Medical Informatics, Biometry und Epidemiology, University Hospital Essen, Essen, Germany; 4 University Hospital Essen, University of Duisburg-Essen, Institute for Urban Public Health, Duisburg, Germany

**Keywords:** Neighbourhood, Social welfare rate, Socio-economic status (SES), Positive affectivity, Older residents

## Abstract

**Background:**

Physical and social neighbourhood characteristics can vary according to the neighbourhood socio-economic status (SES) and influence residents’ perceptions, behaviours and health outcomes both positively and negatively. Neighbourhood SES has been shown to be predictive of mental health, which is relevant for healthy ageing and prevention of dementia or depression. Positive affectivity (PA) is an established indicator of mental health and might indicate a positive emotional response to neighbourhood characteristics. In this study, we focussed on the association of neighbourhood SES with PA among older residents in Germany and considered social integration and environmental perceptions in this association.

**Methods:**

We used questionnaire-based data of the ongoing population-based Heinz Nixdorf Recall Study for our cross-sectional analysis, complemented by secondary data on social welfare rates in the neighbourhood of residents’ address. PA was assessed using the Positive and Negative Affect Schedule (PANAS) in 2016. Linear regression models were performed to estimate the associations and adjusted for socio-demographic variables.

**Results:**

Higher social welfare rates were associated with lower PA scores. The strongest negative association from the crude model (b = −1.916, 95%-CI [−2.997, −0.835]) was reduced after controlling for socio-demographic variables (b = −1.429, 95%-CI [−2.511, −0.346]). Social integration factors (b = −1.199, 95%-CI [−2.276, −0.121]) and perceived environmental factors (b = −0.875, 95%-CI [−1.971, 0.221]) additionally diminished the association of social welfare rates with PA in the full model (b = −0.945, 95%-CI [−2.037, 0.147]).

**Conclusion:**

Our results suggest that neighbourhoods have an influence on the occurrence and the extent of PA. Public health interventions that address socio-economic disadvantage in the neighbourhood environment could be an effective and far-reaching way to reduce the risk of depression and depressive symptoms due to low PA in older residents.

**Supplementary Information:**

The online version contains supplementary material available at 10.1186/s12877-022-03459-9.

## Introduction

### Background

Residents’ neighbourhood environment has been linked to a vast range of health outcomes [1, 2]. Physical and social characteristics of a neighbourhood can influence peoples’ perceptions, behavioural responses and health sequelae both positively and negatively [3]. Physical characteristics relate to health determinants like leisure activities, public transport links, grocery stores and environmental pollution [1]. Social neighbourhood characteristics include neighbourly trust, social networks, social norms and public safety [1, 4]. Both physical and social characteristics have been reported to vary by neighbourhood socio-economic status (SES) [3]. In other words, neighbourhoods that are home to people with migrant backgrounds, to poor or unemployed people and therefore described as ‘low SES neighbourhoods’ often display less favourable physical and social characteristics [3, 5]. Thus, those who need to rely on neighbourly resources have to deal with the least healthy neighbourhood characteristics [6]. In social epidemiological neighbourhood studies, neighbourhood SES has been considered as an indicator of cumulative social and / or physical disadvantage [1, 7].

In addition, the neighbourhood is an important environment especially for older residents: due to retirement and limited mobility, they leave their neighbourhood less often [1, 4]. At the same time, they are becoming an increasingly relevant group for disease prevention and health promotion in aging societies like Germany. Due to demographic change in western Europe, the link between mental and physical health, which becomes stronger with increasing age, is of particular importance. Physical and functional limitations can be both risk factors and consequences of mental illness. Mental health is an essential prerequisite for maintaining everyday competence, social participation and a high quality of life, and its preservation is therefore highly relevant [8].

Neighbourhood SES has been shown to be predictive of mental health. Potential mechanisms linking neighbourhoods to mental health involve physical neighbourhood characteristics like noise, littering, run-down-houses and walkability as well as social characteristics like residents’ social integration [1, 9]. The lack of trustful and personal relationships and the chronic exposure to neighbourhood disorder, vandalism or noise and the perceptions thereof are associated with mental health [1, 9]. Socially disadvantaged people in particular are dependent on supportive (neighbourly) contacts, while often having less contacts at their disposal [10]. Social integration helps to cope with stress, protects against the feeling of isolation and has a positive effect on mental health [1, 9, 11]. If the residential environment does not encourage outdoor activities and the condition of apartments are perceived as poor, this can have negative effects on mental health [1, 3].

Perceptions of environmental stressors and the unavailability of physical and social resources can make people less physically active and less socially involved. However, activity and involvement are important factors of physical and mental health. Moreover, mental health is relevant for healthy aging, prevention of dementia or depression and for coping with other chronic diseases [1, 9, 12]. Barnett et al. (2018) also point out in their systematic review and meta-analysis that the physical and social characteristics of neighbourhoods can influence the extent to which mental illnesses occur in older residents. But not only the existence of negative conditions and events, also the lack of positive experiences in neighbourhoods can lead to mental illnesses [4].

Although the World Health Organisation (WHO) defined health as “not merely the absence of disease and infirmity” [13], health research tends to focus on a pathogenic perspective. This applies to neighbourhood studies, as well. Adopting the comprehensive notion of the WHO, we refer to positive affectivity (PA) as a positive emotional health response to neighbourhood characteristics. PA is characterized by, for example, emotional states like enthusiasm, happiness and excitement [14]. While being valuable for its own sake as a positive mind state and (pre-)condition of the social and mental health dimension, PA has been suggested to protect against chronic diseases (dementia, cardiovascular diseases) [15]. PA is often conceptualised together with negative affectivity (NA). High NA and low PA are part of depression and anxiety disorders [16]. However, PA and NA are not regarded as opposite poles [14, 17]. Rather, PA and NA can co-exist [4], as they are differentiable dimensions [14, 17], each with a distinct psychobiological pattern [15]. Furthermore, emotional states have been conceptualised as bridging mechanism between social and health inequalities [18, 19]. However, to the best of our knowledge, social variations of PA have been rarely investigated in the neighbourhood context.

### Study objective

In this cross-sectional study, we aimed to address this research gap using data from a population-based study on older residents in the Ruhr Area, Germany. The Ruhr Area is an intriguing setting for studies concerned with mental health, as it is characterised by socio-spatial differences in environmental qualities [20–23], population dynamics in combination with general aging trends [24] and a comparatively high burden of disease [25]. The effects of unemployment and socio-economic disadvantage in the neighbourhood context can lead to increased morbidity and mortality, which has so far been conceptualised particularly for the investigation of cardiovascular and metabolic diseases and depressive symptoms in this area [3]. In this line, we examined whether neighbourhood social welfare rate (as a proxy for neighbourhood SES) was inversely associated with PA after accounting for individual sociodemographic and socio-economic characteristics. Moreover, we were interested in two explanatory mechanisms possible relevant for the association between neighbourhood SES and PA, i.e. social integration and environmental perceptions. Thus, we analysed correlations between neighbourhood social welfare rate and social integration and environmental perception variables. Further, we investigated whether these potential mediators statistically explained the association between neighbourhood social welfare rate and PA. Time points of data collection did not allow to perform a mediation analysis, but were close enough in time to construct a cross-sectional data set for this exploratory analysis (see next paragraph).

**Fig. 1:**
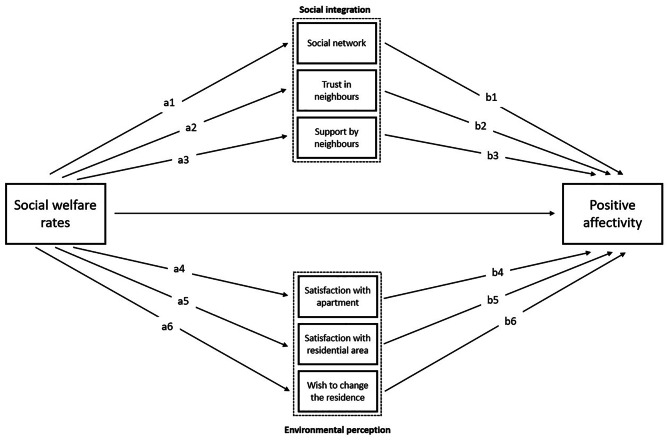
Figure 1 illustrates our research objective focussing on the association of neighbourhood SES with PA. Conceptually, social integration and environmental perception variables act as mediators in this association. The associations a1 – a6 are approximated by a correlation analysis, the associations b1 – b6 are assessed by a regression analysis

## Methods

### Study population

**Fig. 2:**
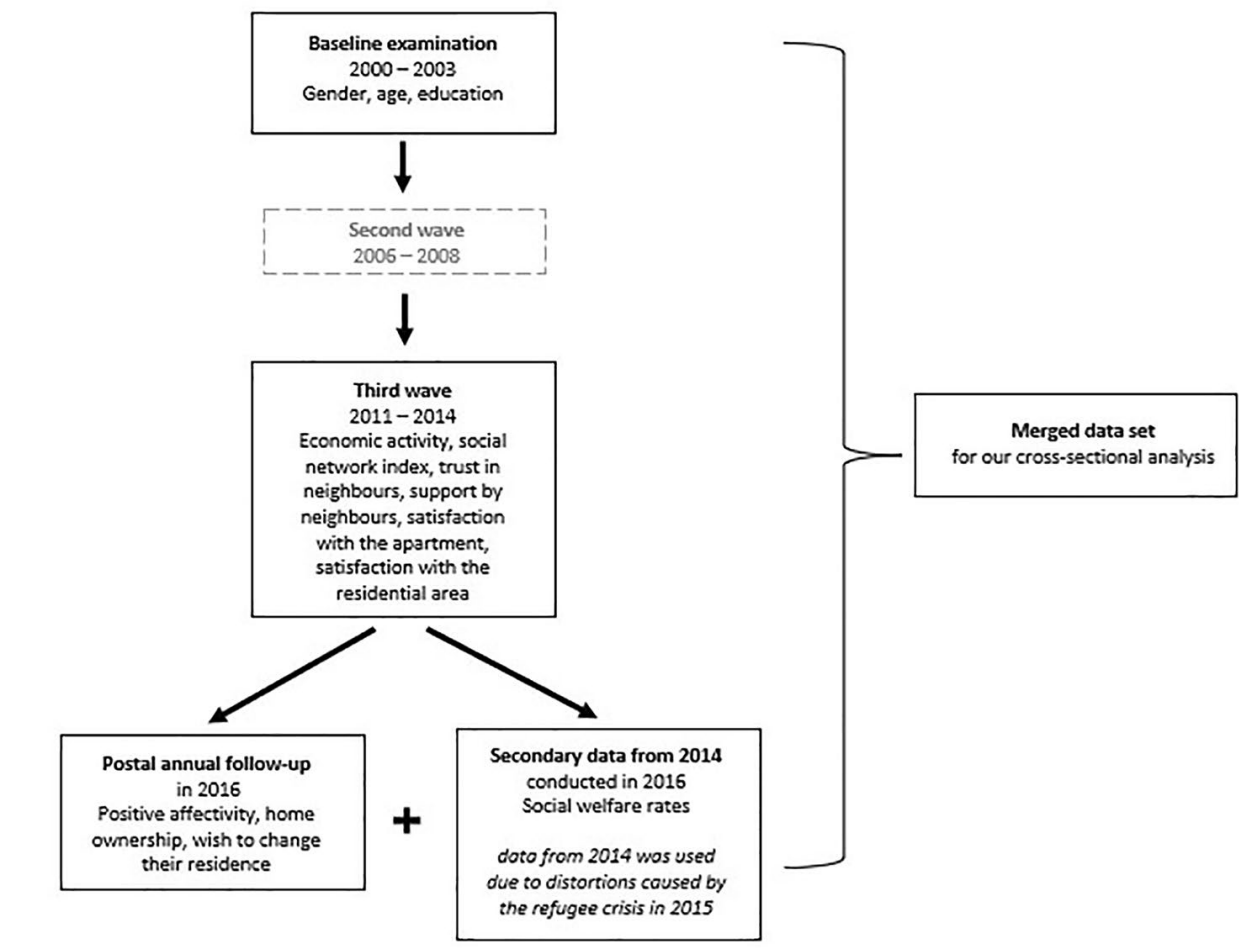
Figure 2 shows the origin of variables used to build the data set four our cross-sectional analysis. Information on gender, age and education were taken from the baseline examination and considered as rather stable in this study sample. All other variables stemmed from the third wave and the postal annual follow-up, reflecting the most recent information. Therefore, no variables were retrieved from the second wave

The data merged for this cross-sectional study was collected during baseline examination (2000–2003), the third wave (2011–2014) and the postal annual follow-up in 2016 of the ongoing prospective population-based Heinz Nixdorf Recall Study (HNR), as shown in Fig. 2. This study is being conducted in three major cities of the Ruhr Area (Bochum, Essen, Mülheim / Ruhr) in western Germany. Rationale and study design have been described in detail elsewhere [26]. The entire cohort of the HNR study comprised 4814 participants at baseline (50.2% omen, aged 45–75 years). 2899 participants were considered as still active at the time of the annual follow-up in 2016. Reasons for dropping out were mainly ill-health and death or residential re-locations out of the study region. Around 83% responded to this postal survey that included an additional questionnaire on “Traffic Noise and Well-being” (N = 2406). After merging the data from baseline examination, the third wave and from the postal survey, missing data on exposure, outcome and all covariates were excluded (N = 1861). Since data on exposure is from 2014 and the outcome data is from 2016, the number of participants was limited to those who had lived at their current home address in 2016 for at least two years. Thus, the final sample size was N = 1768.

### Exposure measure

As a proxy for neighbourhood SES, the mean social welfare rate within a radius of 500 m of participants’ home addresses was used. The social welfare rate is based on the German social code and provides basic income security, especially in the case of long-term unemployment [27]. Data on social welfare rates were provided by the city administrations of Bochum, Essen and Mülheim/Ruhr. Due to distortions caused by the refugee crises in 2015, data from 2014 was used (see Fig. 2). Measured for small-scale statistical units in 2014 (median inhabitants per unit: 1736 in Bochum, 1771 in Essen and 1496 in Mülheim/Ruhr), this variable was weighted by the area proportions of statistical units within this radius. Because of different social welfare rate levels in the three cities, participants’ neighbourhood social welfare rate was categorised into city-specific quartiles, using the first quartile (lowest welfare rates) as reference category. In small-scale social monitoring, the social welfare rate is often used as an indicator to depict the socio-economic situation of a neighbourhood or to identify and describe areas for urban regeneration projects [27].

### Outcome measure

PA was measured by using the “Positive and Negative Affect Schedule” (PANAS) [17], a frequently used validated questionnaire in self-reported mental health assessments. It contains 10 items for PA and 10 items for NA, each of them rated on a 5-point Likert scale of 1 (not at all) to 5 (extremely), yielding a potential sum score from 10 to 50. For PA, participants indicated to what extent they felt, i.e. enthusiastic, happy or excited.

Cronbach’s alpha was calculated to assess the internal consistency of the subscale for PA in this study sample. The internal consistency was satisfying, with Cronbach’s alpha = 0.89.

### Socio-demographic variables (potential confounders)

Socio-demographic factors like female gender, older age, unemployment, low educational status and living for rent are considered as risk factors for mental health [1, 11, 28]. At the same time, they could have determined participants’ residential location [29].

Gender (binary), age in 2016 and SES variables (economic activity in 2014, education and housing situation) were included in the sociodemographic model (Statistical analyses). Economic activity was classified into four groups (“employed”, “inactive/ homemaker”, “retired”, “unemployed”). Years of formal education and vocational training were defined according to the International Standard Classification of Education (ISCED) [30], and categorised into four groups (“≤ 10 years”, “11–13 years”, “14–17 years”, “≥ 18 years”) [31]. Participants’ status on home ownership was categorised in two groups: “property” and “for rent”.

### Social integration variables (potentially explanatory variables)

The social network index was built according to Berkman’s social integration and health model [32]. It includes marital status (“single”, “divorced”, “widowed”, “married”, “living with a partner”), social ties and frequency of contacts with close friends and family, number of close persons you see at least once a month and engagement or membership in organizations. An index was then built from these individual variables and divided into four categories (“large”, “medium”, “small”, “social isolation”) [10]. Trust in neighbours (e.g. “I can trust most people in my neighbourhood”) and support by neighbours (e.g. “Most people in my neighbourhood are willing to help”) were measured by a 4-point Likert scale from 1 (“fully disagree”) to 4 (“fully agree”) [21] and divided into four categories (“poor”, “average”, “good”, “excellent”). As the number of cases in the categories “poor” and “average” was low, these two categories were combined (“poor to average”). These variables all relate to the participants’ social integration and may vary by neighbourhood SES.

### Environmental perception variables (potentially explanatory variables)

Participants were asked to rate their satisfaction with the apartment/ house (“How satisfied are you with your apartment?”) and with the residential area (“How satisfied are you with your residential area?”), both measured by a 4-point Likert scale from 1 (“very unsatisfied”) to 4 (“very satisfied”) [21] and divided into four categories (“poor”, “average”, “good”, “excellent”). As the number of cases in the categories “poor” and “average” was low, these two categories were combined (“poor to average”). Participants were also asked whether they wish to change their residence (“Would you like to move?” yes/no) [33]. These variables all relate to how participants perceive their neighbourhood environment.

### Statistical analyses

Sample characteristics were described using absolute and relative frequencies and means with standard deviations. Correlations between neighbourhood social welfare rate and explanatory variables were analysed according to their scale (binary or ordinal) and assessed following Cohen’s recommendation (1988). Bivariate and multivariate linear regression models were used to determine the associations between neighbourhood social welfare rates and PA. A series of models were built to estimate the associations between neighbourhood social welfare and the outcome variable (PA):

Model 1 - “crude”: without additional variables.

Model 2 - “socio-demographic model”: model 1 + socio-demographic variables (gender, age,

education, home ownership, economic activity).

Model 3 - “social integration model”: model 2 + social integration variables (strength of.

participants social network, trust in neighbours, support by neighbours).

Model 4 - “environmental perception model”: model 2 + environmental perception variables (participants satisfaction with the apartment or the residential area, wish to change their residence).

Model 5 - “full model”: model 2 + social integration + environmental perception variables.

In previous analyses, the linear regression models were stratified by gender and extended with interaction terms to test for effect modification. Since we found no differences in the pattern between females and males and the interaction terms were not nearly significant, we decided not to stratify the analyses by gender.

SAS® 9.4 (TS1M6) was used to perform all statistical analyses (SAS Institute Inc., Car, NC, USA).

### Sensitivity analyses - neighbourhood radius 300 m instead of 500 m

Since the radius, as described in Exposure measure, is only an approximation for a neighbourhood and neighbourhoods can be defined in different ways, all analyses were done again with a smaller radius around the residential addresses (300 m).

## Results

### Sample characteristics

All 1768 participants included in the complete case analysis are presented in Table 1. Most participants lived in neighbourhoods with medium social welfare rates (mid-low = 34.4% and mid-high = 29.0%), the fewest in neighbourhoods with the highest social welfare rates (11.4%). Gender was quite balanced (50.9% women) and the mean age of the entire study population was 71.24 (SD = 6.87). More than the half of participants had 11–13 years of education (55.3%) and lived in property (57.9%). 67.6% were retired and 67.0% had a medium-sized social network. Most participants reported good trust in neighbours (52.0%), good (47.6%) or excellent (45.1%) support by neighbours and rated the satisfaction with their apartment as excellent (71.8%), as well as the satisfaction with their residential area (59.6%). Overall, 92.6% did not want to change their residence.

**Table 1:** Characteristics of the complete case sample

(N = 1768)	Number (%) or mean [SD]	Positive affectivity score
**Social welfare rate within 500 m**		
Low	446 (25.2)	34.38 [6.34]
Mid-low	608 (34.4)	33.61 [6.31]
Mid-high	513 (29.0)	33.43 [6.60]
High	201 (11.4)	32.46 [7.04]
**Social welfare rate within 300 m**		
Low	494 (27.9)	34.46 [6.36]
Mid-low	569 (32.2)	33.60 [6.29]
Mid-high	469 (26.5)	33.29 [6.60]
High	236 (13.4)	32.56 [6.94]
**Positive affectivity score**		
Mean score of the entire sample	/	33.62 [6.51]
**Gender**		
Female	900 (50.9)	33.39 [6.55]
Male	868 (49.1)	33.86 [6.46]
**Age. range 60–90**		
Increasing age	71.24 [6.87]	/
**Education (ISCED)**		
≤ 10 years	106 (6.0)	31.42 [6.52]
11–13 years	978 (55.3)	33.19 [6.79]
14–17 years	442 (25.0)	34.20 [5.99]
≥ 18 years	242 (13.7)	35.27 [5.74]
**Home ownership**		
Yes	1024 (57.9)	34.33 [6.30]
No	744 (42.1)	32.64 [6.67]
**Economic activity**		
Employed	423 (23.9)	34.99 [6.06]
Inactive/ Homemaker	88 (5.0)	33.79 [6.86]
Retired	1197 (67.7)	33.19 [6.57]
Unemployed	60 (3.4)	32.35 [6.51]
**Social network index**		
Large	67 (3.8)	36.21 [6.32]
Medium	1184 (67.0)	34.07 [6.40]
Small	469 (26.5)	32.35 [6.53]
“Social isolation“	48 (2.7)	31.35 [6.71]
**Trust in neighbours**		
Poor to average	207 (17.7)	31.73 [6.82]
Good	920 (52.0)	33.19 [6.08]
Excellent	641 (36.3)	34.85 [6.77]
**Support by neighbours**		
Poor to average	129 (7.3)	30.91 [6.90]
Good	842 (47.6)	33.08 [6.19]
Excellent	797 (45.1)	34.63 [6.58]
**Satisfaction with the apartment**		
Poor to average	52 (2.9)	31.51 [6.85]
Good	447 (25.3)	31.06 [5.97]
Excellent	1269 (71.8)	34.61 [6.41]
**Satisfaction with the residential area**		
Poor to average	76 (4.3)	32.22 [6.67]
Good	639 (36.1)	32.22 [6.44]
Excellent	1053 (59.6)	34.57 [6.37]
**Wish to change their residence**		
Yes	131 (7.4)	32.61 [6.23]
No	1637 (92.6)	33.70 [6.52]

Mean PA score of the entire study population was 33.62 (SD = 6.51). PA scores in neighbourhoods with high social welfare rates were lower than in the other groups (32.46; SD = 7.04). Home ownership, employment and high education (18 or more years) showed higher PA scores than in the other related categories. Participants with a large social network had the highest PA score in the entire study sample (36.21; SD = 6.32). Participants with good or excellent trust in or support by neighbours scored higher on the PA scale than those, who reported poor to average trust or support. Same applies to those who rated the satisfaction with their apartment as excellent: they had higher scores of PA than those in the ‘poor to average’ group.

### Correlation of neighbourhood social welfare rates with potential mediator variables

As presented in Table 2, correlation analyses showed that neighbourhood social welfare rates and the social network index were not correlated with each other (r = 0.01). Higher social welfare rates in the neighbourhood were correlated with participants’ assumption of reduced trust in and support by neighbours (r = −0.18 and r = −0.13).

**Table 2:** Correlation analyses of neighbourhood social welfare rates with potential mediator variables

	Social network index	Trust in neighbours	Support by neighbours	Satisfaction with the apartment	Satisfaction with the residential area	Wish to change the residence¹
**Social welfare rate within 500 m**	0.007 p = 0.768	−0.184 p < 0.0001	−0.132 p < 0.0001	−0.130 p < 0.0001	−0.303 p < 0.0001	0.059 p = 0.013

¹ Pearson correlation analysis was used for this dichotomous variable. All other variables were calculated with Spearman

There was a weak negative correlation between neighbourhood social welfare rates and satisfaction with the housing situation(r = −0.13), while satisfaction with the living environment showed a moderate negative correlation (r = −0.30). The participants’ wish to change the residence was not correlated with the neighbourhood social welfare rate (r = 0.06).

### Association of neighbourhood social welfare rates with positive affectivity

Results of linear regression models on the association of neighbourhood social welfare rates with PA are presented in Table 3. In the crude linear regression model 1, a negative association between social welfare rates and PA was observed, with effect sizes increasing from the lowest to the highest quartile. This means, residents living in neighbourhoods in the highest social welfare quartile had a PA score almost 2 points lower than those in the lowest quartile.

**Table 3:** Results from linear regression analyses of neighbourhood social welfare rate with positive affectivity

	Model 1 “crude”			Model 2 “socio-demographics”			Model 3 “social integration”			Model 4 “environmental perception”			Model 5 “full model”		
**Exposure variable**															
**SGBII 500 m**	**b**	**95%-CI**		**b**	**95%-CI**		**b**	**95%-CI**		**b**	**95%-CI**		**b**	**95%-CI**	
Lowest	Ref.	/	/	Ref.	/	/	Ref.	/	/	Ref.	/	/	Ref.	/	/
Mid-low	−0.764	−1.557	0.030	−0.544	−1.328	0.239	−0.392	−1.168	0.384	−0.268	−1.037	0.502	−0.227	−0.992	0.538
Mid-high	−0.951	−1.775	−0.127	−0.508	−1.332	0.317	−0.239	−1.059	0.581	0.013	−0.810	0.836	0.054	−0.766	0.873
Highest	−1.916	−2.997	−0.835	−1.429	−2.511	−0.346	−1.199	−2.276	−0.121	−0.875	−1.971	0.221	−0.945	−2.037	0.147
**Socio-demographic variables**															
**Gender**															
Female				−0.046	−0.693	0.602	−0.036	−0.694	0.622	−0.046	−0.677	0.585	0.032	−0.614	0.677
Male				Ref.	/	/	Ref.	/	/	Ref.	/	/	Ref.	/	/
**Age**															
Increasing age				−0.101	−0.156	−0.045	−0.098	−0.153	−0.043	−0.119	−0.173	−0.065	−0.112	−0.166	−0.057
**Education (ISCED)**															
≤ 10 years				−2.022	−3.589	−0.456	−1.648	−3.195	−0.100	−1.891	−3.418	−0.363	−1.641	−3.160	−0.122
11–13 years				−1.192	−2.147	−0.237	−1.026	−1.970	−0.083	−1.259	−2.189	−0.329	−1.133	−2.058	−0.208
14–17 years				−0.520	−1.538	0.497	−0.340	−1.346	0.665	−0.435	−1.428	0.557	−0.326	−1.314	0.661
≥ 18 years				Ref.	/	/	Ref.	/	/	Ref.	/	/	Ref.	/	/
**Home ownership**															
Yes				Ref.	/	/	Ref.	/	/	Ref.	/	/	Ref.	/	/
No				−1.131	−1.770	−0.492	−0.716	−1.356	−0.077	−0.532	−1.169	0.104	−0.273	−0.911	0.365
**Economic activity**															
Employed				Ref.	/	/	Ref.	/	/	Ref.	/	/	Ref.	/	/
Inactive/ Homemaker				−0.710	−2.223	0.802	−0.834	−2.332	0.665	−0.843	−2.316	0.630	−0.974	−2.443	0.494
Retired				−0.615	−1.485	0.254	−0.554	−1.412	0.304	−0.599	−1.446	0.247	−0.560	−1.400	0.281
Unemployed				−2.126	−3.860	−0.392	−1.993	−3.704	−0.283	−1.775	−3.464	−0.086	−1.688	−3.364	−0.011
**Social integration variables**															
**Social network index**															
Large							Ref.	/	/				Ref.	/	/
Medium							−1.698	−3.257	−0.138				−1.847	−3.376	−0.318
Small							−2.744	−4.387	−1.102				−2.812	−4.425	−1.200
“Social isolation”							−3.862	−6.224	−1.501				−3.890	−6.202	−1.577
**Trust in neighbours**															
Poor to average							−0.993	−2.356	0.369				−0.596	−1.940	0.748
Good							−0.737	−1.605	0.130				−0.499	−1.353	0.355
Excellent							Ref.	/	/				Ref.	/	/
**Support by neighbours**															
Poor to average							−2.379	−3.902	−0.856				−1.860	−3.359	−0.361
Good							−0.887	−1.726	−0.047				−0.581	−1.407	0.245
Excellent							Ref.	/	/				Ref.	/	/
**Environmental perception variables**															
**Satisfaction with the apartment**															
Poor to average										−2.881	−4.916	−0.847	−2.537	−4.561	−0.513
Good										−2.965	−3.766	−2.164	−2.899	−3.695	−2.104
Excellent										Ref.	/	/	Ref.	/	/
**Satisfaction with the residential area**															
Poor to average										0.182	−1.578	1.942	0.736	−1.025	2.497
Good										−0.882	−1.616	−0.147	−0.491	−1.234	0.252
Excellent										Ref.	/	/	Ref.	/	/
**Wish to change the residence**															
Yes										−0.167	−1.322	0.987	−0.049	−1.198	1.099
No										Ref.	/	/	Ref.	/	/

After accounting for socio-demographic variables in model 2, the negative association diminished. Estimated effects for the second and third quartile of neighbourhood social welfare became similar and weaker, with upper confidence intervals including zero, whereas estimates for the fourth quartile still indicated a negative association (b = −1.429, 95%-CI [−2.511, −0.346]). In the subsequent models 3–5, estimated effects were further reduced. Having included the social integration variables to the socio-demographic model (model 3), the strongest negative association was shown in the fourth quartile (b = − 1.199, 95%-CI [−2.276, −0.121]). The smaller the social network and the poorer the support by neighbours, the lower was the PA score. In model 4, environmental perception variables were added to the socio-demographic model 2 and the negative association in the fourth quartile was weakened (b = − 0.875, 95%-CI [−1.971, 0.221]). Among perceived environmental factors, satisfaction with the apartment yielded the most distinct association with PA. In the full model 5, the coefficient in the fourth quartile became slightly stronger (b = − 0.945, 95%-CI [−2.037, 0.147]) compared to the environmental perception model (b = −0.875, 95%-CI [−1.971, 0.221]), but the width of the confidence intervals was rather similar in both models.

### Sensitivity analyses - neighbourhood radius 300 m instead of 500 m

Sensitivity analyses showed similar patterns as the main analyses, associations were slightly weaker, except in model 4 (supplementary S1). Correlation coefficients were also similar but a little weaker as in the main analyses.

## Discussion

This study investigated possible associations between neighbourhood social welfare rates and PA among older residents. We hypothesized that higher social welfare rates may lead to lower scores on PA and that social integration and environmental perception variables may be mediators in this association.

As hypothesized, the results of this cross-sectional regression analysis showed that higher social welfare rates in the neighbourhood were linked to lower PA scores, in contrast to the reference group (lowest social welfare rates), when controlled for socio-demographic characteristics and when social integration factors were included in the model. The negative association decreased across the models, in particular after including the perceived environmental factors. The associations in the fourth quartiles proved to be the most consistent across the models. All social integration variables produced associations in the expected directions, with PA being lower if the social networks, neighbourhood trust and support were less pronounced (Table 3). Among environmental perception variables – mutually adjusted for gender, age, education, home ownership and economic activity – satisfaction with the apartment was the strongest predictor of PA, which might emphasise the psychological meaning of home. Previous studies found that neighbourhood SES is related to the individual’s adaptiveness to adverse conditions by affecting the exposure to environmental stressors and the availability of physical and social resources in the neighbourhood [1]. Neighbourhood characteristics like noise, littering, run-down-houses and disorder are important influencing factors for mobility and mental health in older adults, but social integration is also very important [1, 3, 9, 34, 35]. This is because socially disadvantaged and older people in particular are dependent on the supportive functions of a neighbourhood. Social ties often diminish with age and the radius of action is mostly limited to the immediate surroundings. Neighbourhood contacts can promote participation in societal life, both through general inclusion and through support, especially in health crises [36]. Generally, social factors are considered as stronger determinants for mental health than environmental factors [1, 4]. But it is also possible that environmental factors and social factors are mutually dependent. A negative neighbourhood environment in which people do not like to move around freely may not invite to have social contacts outside the apartment. Adequate public places could lead to a positive association between the neighbourhood environment and social integration [4, 21, 35, 37]. The more comfortable older residents feel in their neighbourhood, the more they walk by foot, the longer they stay mobile [37] and the more likely they can draw resources from their neighbourhood (incl. local embeddedness of contacts, trust in neighbours etc.).

In addition, neighbourhood SES was negatively correlated with neighbourhood trust and support as well as with satisfaction with the apartment and with the residential area. This might support the conceptual idea of social integration and environmental perceptions being partial mediators. However, we could not observe a correlation of neighbourhood social welfare rate with the social network index. Not restricted to the neighbourhood according to the measurement instructions, the social network index appeared to be an indicator of social integration that was not affected by neighbourhood SES, but was predictive of PA (Table 3). Thus, the components of the social network index could point to inter-individual intervention needs in their own right – in addition to neighbourhood needs.

Some studies suggest that there may be a mixed effect between individual SES and neighbourhood SES. In Stafford and Marmot’s (2003) study, neighbourhood disadvantage was associated with health outcomes beyond individual SES. Depending on employment grade, people who lived in disadvantaged neighbourhoods had poorer health status than people who lived in less disadvantaged neighbourhoods. The impact of the residential environment on general and mental health appeared to be greater among people with low individual SES. They also reported more neighbourhood problems [38]. Nguyen et al. (2013) found no impact on individual SES when moving from a high poverty neighbourhood to a low poverty neighbourhood. However, participants’ mental health improved [39]. Pickett and Pearl (2001) point out that analyses must adequately account for individual SES, otherwise neighbourhood-level effects could serve as proxies for unmeasured aspects of individual SES. Since neighbourhood SES can determine a person’s income, education and occupation, controlling for individual SES could cancel out some of the context effect. Measures of neighbourhood SES can therefore be seen as proxies for both unmeasured mechanisms and actual exposure per se, or both [40].

Some limitations have to be considered. One limitation could be the self-completed questionnaire in relation to assess PA. Older people tend to give more extreme answers in both directions [41]. Mental health can be considered as a sensitive topic and older people in particular may have a different attitude towards mental health [42]. Therefore, social desirability should be taken into account [43] which may have led to an underestimation of associations for PA. Furthermore, the study sample is a highly selective group, as they already belong to the healthier participants and survivors of a cohort, which may have led to a selection bias. Regarding external validity, the results are not generalisable to other population groups (e.g. younger people, people with a migrant background) due to the demographic structure of our cohort.

General strengths of this study include the usage of a large sample size and that data for this cross-sectional study was taken from a population-based longitudinal study. To the best of our knowledge, this is also the first study that examines PA in relation to neighbourhood social welfare rates among older residents. The outcome was assessed by a validated questionnaire which has been shown to well-reflect PA. Results of this study can be considered as robust in terms of two radius-based neighbourhood delineations, as the sensitivity analyses came to similar results.

## Conclusion

According to the results of this study, higher levels of social welfare rates in the neighbourhood are associated with a decrease in PA. These results suggest that neighbourhoods have an influence on the occurrence and extent of PA, which in turn is important for mental health and healthy ageing in older residents. Future research should therefore continue to identify both neighbourhood-related and (inter-)individual factors that lead to an increase or decrease of PA. Public health interventions that address socio-economic disadvantage in the neighbourhood environment could be an effective and far-reaching way to reduce the risk of depression and depressive symptoms due to low PA in older residents. Furthermore, a deeper examination of the gender-specific contribution of the confounding and potentially mediating variables could yield interesting insights. The INGER project (“Integrating gender into environmental health research”) has developed a comprehensive basis for this with the multidimensional INGER sex/gender concept [44].

## Electronic supplementary material

Below is the link to the electronic supplementary material.

Supplementary Material: Table S1: Results from linear regression analyses of neighbourhood social welfare rate (300 meters instead of 500)

## Data Availability

The datasets generated and analysed during the current study are not publicly available due to data protection rules in Germany, but are available for researchers who meet the criteria of the University Hospital Essen for access to confidential data upon justified request to the corresponding author.

## Declarations

### Accordance with guidelines

The authors adhered to the STROBE guidelines for reporting research (www.equator-network.org/reporting-guidelines/strobe/).
