# Supplementary material for: Neighbourhood socio-economic status and positive affectivity among older residents in Germany: a cross-sectional analysis with data from the Heinz Nixdorf Recall Study
Source: BMC Geriatr. 2022 Nov 22;22:891. doi: 10.1186/s12877-022-03459-9 (PMC9682725; doi:10.1186/s12877-022-03459-9)
Supplement: Supplementary file 1 — Supplementary Material: Table S1: Results from linear regression analyses of neighbourhood social welfare rate (300 meters instead of 500) [file 12877_2022_3459_MOESM1_ESM.docx]

**Supplement Table S1: Results from linear regression analyses of neighbourhood social welfare rate (300 meters instead of 500)**

|  | **Model 1**  **“crude”** | | | **Model 2**  **“socio-demographics”** | | | **Model 3**  **“social integration”** | | | **Model 4**  **“environmental perception”** | | | **Model 5**  **“full model”** | | |
| --- | --- | --- | --- | --- | --- | --- | --- | --- | --- | --- | --- | --- | --- | --- | --- |
| **Exposure variable** | | | | | | | | | | | | | | | |
| **SGBII 300m** | **b** | **95%-CI** | | **b** | **95%-CI** | | **b** | **95%-CI** | | **b** | **95%-CI** | | **b** | **95%-CI** | |
| Lowest | Ref. | / | / | Ref. | / | / | Ref. | / | / | Ref. | / | / | Ref. | / | / |
| Mid-low | -0.866 | -1.648 | -0.085 | -0.602 | -1.375 | 0.171 | -0.485 | -1.248 | 0.279 | -0.415 | -1.173 | 0.342 | -0.376 | -1.128 | 0.377 |
| Mid-high | -1.168 | -1.988 | -0.349 | -0.682 | -1.505 | 0.140 | -0.461 | -1.277 | 0.354 | -0.256 | -1.074 | 0.562 | -0.236 | -1.049 | 0.577 |
| Highest | -1.899 | -2.905 | -0.893 | -1.359 | -2.370 | -0.348 | -1.128 | -2.133 | -0.122 | -0.922 | -1.943 | 0.100 | -0.952 | -1.969 | 0.066 |
| **Socio-demographic variables** | | | | | | | | | | | | | | | |
| **Gender** |  |  |  |  |  | |  |  | |  |  | |  |  | |
| Female |  |  |  | -0.038 | -0.686 | 0.609 | -0.028 | -0.686 | 0.631 | -0.039 | -0.671 | 0.593 | 0.040 | -0.606 | 0.686 |
| Male |  |  |  | Ref. | / | / | Ref. | / | / | Ref. | / | / | Ref. | / | / |
| **Age** |  |  |  |  |  |  |  |  |  |  |  |  |  |  |  |
| Increasing age |  |  |  | -0.100 | -0.155 | -0.045 | -0.097 | -0.153 | -0.042 | -0.119 | -0.173 | -0.065 | -0.112 | -0.166 | -0.057 |
| **Education (ISCED)** |  |  |  |  |  |  |  |  |  |  |  |  |  |  |  |
| ≤ 10 years |  |  |  | -1.976 | -3.543 | -0.410 | -1.599 | -3.148 | -0.051 | -1.826 | -3.354 | -0.298 | -1.578 | -3.097 | -0.058 |
| 11 – 13 years |  |  |  | -1.159 | -2.114 | -0.204 | -0.991 | -1.934 | -0.048 | -1.217 | -2.147 | -0.287 | -1.091 | -2.016 | -0.166 |
| 14 – 17 years |  |  |  | -0.488 | -1.505 | 0.529 | -0.307 | -1.312 | 0.698 | -0.395 | -1.387 | 0.597 | -0.288 | -1.274 | 0.699 |
| ≥ 18 years |  |  |  | Ref. | / | / | Ref. | / | / | Ref. | / | / | Ref. | / | / |
| **Home ownership** |  |  |  |  |  |  |  |  |  |  |  |  |  |  |  |
| Yes |  |  |  | Ref. | / | / | Ref. | / | / | Ref. | / | / | Ref. | / | / |
| No |  |  |  | -1.109 | -1.749 | -0.468 | -0.697 | -1.338 | -0.056 | -0.506 | -1.144 | 0.132 | -0.250 | -0.890 | 0.390 |
| **Economic activity** |  |  |  |  |  |  |  |  |  |  |  |  |  |  |  |
| Employed |  |  |  | Ref. | / | / | Ref. | / | / | Ref. | / | / | Ref. | / | / |
| Inactive/ Homemaker |  |  |  | -0.746 | -2.257 | 0.765 | -0.865 | -2.362 | 0.633 | -0.871 | -2.343 | 0.602 | -1.003 | -2.471 | 0.465 |
| Retired |  |  |  | -0.618 | -1.487 | 0.252 | -0.554 | -1.413 | 0.304 | -0.600 | -1.447 | 0.247 | -0.560 | -1.400 | 0.281 |
| Unemployed |  |  |  | -2.074 | -3.810 | -0.339 | -1.945 | -3.657 | -0.233 | -1.728 | -3.419 | -0.038 | -1.639 | -3.318 | 0.039 |
| **Social integration variables** | | | | | | | | | | | | | | | |
| **Social network index** |  |  |  |  |  |  |  |  |  |  |  |  |  |  |  |
| Large |  |  |  |  |  |  | Ref. | / | / |  |  |  | Ref. | / | / |
| Medium |  |  |  |  |  |  | -1.687 | -3.246 | -0.127 |  |  |  | -1.850 | -3.379 | -0.321 |
| Small |  |  |  |  |  |  | -2.732 | -4.375 | -1.089 |  |  |  | -2.811 | -4.424 | -1.198 |
| “Social isolation” |  |  |  |  |  |  | -3.846 | -6.208 | -1.484 |  |  |  | -3.885 | -6.198 | -1.572 |
| **Trust in neighbours** |  |  |  |  |  |  |  |  |  |  |  |  |  |  |  |
| Poor to average |  |  |  |  |  |  | -1.007 | -2.368 | 0.354 |  |  |  | -0.595 | -1.938 | 0.748 |
| Good |  |  |  |  |  |  | -0.736 | -1.603 | 0.130 |  |  |  | -0.489 | -1.343 | 0.365 |
| Excellent |  |  |  |  |  |  | Ref. | / | / |  |  |  | Ref. | / | / |
| **Support by neighbours** |  |  |  |  |  |  |  |  |  |  |  |  |  |  |  |
| Poor to average |  |  |  |  |  |  | -2.353 | -3.875 | -0.831 |  |  |  | -1.845 | -3.343 | -0.346 |
| Good |  |  |  |  |  |  | -0.868 | -1.707 | -0.028 |  |  |  | -0.567 | -1.393 | 0.259 |
| Excellent |  |  |  |  |  |  | Ref. | / | / |  |  |  | Ref. | / | / |
| **Environmental perception variables** | | | | | | | | | | | | | | | |
| **Satisfaction with the apartment** |  |  |  |  |  |  |  |  |  |  |  |  |  |  |  |
| Poor to average |  |  |  |  |  |  |  |  |  | -2.968 | -5.009 | -0.927 | -2.618 | -4.648 | -0.588 |
| Good |  |  |  |  |  |  |  |  |  | -2.971 | -3.772 | -2.170 | -2.904 | -3.700 | -2.108 |
| Excellent |  |  |  |  |  |  |  |  |  | Ref. | / | / | Ref. | / | / |
| **Satisfaction with the residential area** |  |  |  |  |  |  |  |  |  |  |  |  |  |  |  |
| Poor to average |  |  |  |  |  |  |  |  |  | 0.229 | -1.531 | 1.990 | 0.769 | -0.992 | 2.530 |
| Good |  |  |  |  |  |  |  |  |  | -0.844 | -1.576 | -0.111 | -0.460 | -1.200 | 0.281 |
| Excellent |  |  |  |  |  |  |  |  |  | Ref. | / | / | Ref. | / | / |
| **Wish to change the residence** |  |  |  |  |  |  |  |  |  |  |  |  |  |  |  |
| Yes |  |  |  |  |  |  |  |  |  | -0.164 | -1.317 | 0.990 | -0.048 | -1.195 | 1.100 |
| No |  |  |  |  |  |  |  |  |  | Ref. | / | / | Ref. | / | / |
